# Role of Organizational Resilience and Psychological Resilience in the Workplace—Internal Stakeholder Perspective

**DOI:** 10.3390/ijerph191811799

**Published:** 2022-09-19

**Authors:** Tai-Ming Wut, Stephanie-Wing Lee, Jing (Bill) Xu

**Affiliations:** College of Professional and Continuing Education, The Hong Kong Polytechnic University, Hong Kong, China

**Keywords:** organizational resilience, employee resilience, psychological resilience, perceived well-being, work engagement, internal stakeholder

## Abstract

The role of organizational resilience is important in an era of the new normal after COVID-19. The purpose of this study is to examine the effect of organizational resilience and psychological resilience on perceived well-being and employee resilience in the workplace from the internal stakeholder perspective. A new research framework has been proposed. Cross-sectional research design was employed to collect responses from 115 employees from various organizations. Structural equation modeling was used to analyze the data. Organizational resilience is associated with perceived well-being and employee resilience. Psychological resilience is associated with perceived well-being and employee resilience. Employee resilience and perceived well-being are associated with work engagement. Complex mediation models are proposed. Theoretical contributions and managerial implications are discussed.

## 1. Introduction

Geopolitical threats, new technology, changing demographics, and de-globalization are recent top considerations of corporations worldwide. In order to ensure growth and survival in today’s market environment, organizations need to be more flexible and creative [1]. When examining organizational resilience, the interaction between organization and environment must be investigated. Similarly, research on employee resilience is not complete without studying organization resilience and external threats. We must be prepared for the new normal, i.e., living with COVID-19 for a prolonged period of time, wearing face masks, and maintaining social distance for at least a few more years. The question is, how can employee resilience and work engagement be sustained in the workplace? Organizational resilience has a unique role in employee resilience and work engagement [2]. Previous research findings indicate that learning organization is associated with employee resilience. Owing to organization resilience, a learning organization easily adapts to a rapidly changing environment [3]. Despite this, few studies have examined how organization resilience facilitates employee work engagement in the workplace.

To address this research gap, the purpose of our study was to examine the effect of organizational resilience and psychological resilience on perceived well-being, employee resilience, and work engagement in the workplace. Work engagement was found to be a main determinant of corporate performance. Thus, there is an urgent need for our research. A cross-sectional research design was employed in this study and target respondents were employees in the workplace. Our results showed how psychological resilience and organization resilience affected work engagement. 

The remainder of the study is organized as follows. Our research framework is presented in the literature review section (Section 2). Section 3 concerns the development of research hypotheses, while Section 4 regards the methodology used and the data collection. Our results are presented and discussed in Section 5, while Section 6 concludes our study.

## 2. Literature Review

### 2.1. Resilience

According to Soanes and Stevenson [4] (p. 1498), resilience is defined as being “able to withdraw or recover quickly from difficult conditions” [4]. The term is also expressed as “encompassing positive adaptation within the context of significant adversity” [5] (p. 543). Sources of resilience come from personal factors and environmental factors. Personality traits, emotions, and adaptability are examples of personal factors. Positive emotions have been suggested to enhance resilience. Social support from family and peers was found to be associated with resilience. Good education, community services, sports opportunities, and cultural factors were the environments affecting resilience [6,7].

**Figure 1:**
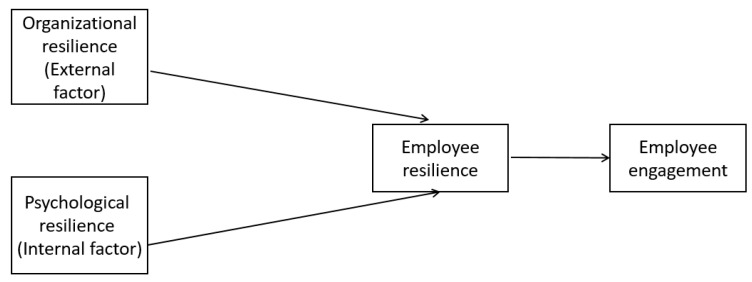
Basic research model (Source: authors).

Our conceptual framework is adapted from Herrman et al.’s [6] notion of employee resilience. Psychological resilience is an internal factor within the individual employee, while organizational resilience is an external environmental factor relative to the employee. These two factors contribute to employee resilience which leads to employees’ work engagement. The basic research model is listed as follows (Figure 1):

### 2.2. Psychological Resilience

The definition of psychological resilience consists of two elements: adversity and positive adaptation. One might say that adversity is an antecedent of resilience and the consequence is positive adaptation. There is a range of adversity from personal work stress to intensive stress. One has to demonstrate their competence to cope with adversity [8]. Psychological resilience details a personal resource to adapt in adverse situations [9]. It has an important role in coping with stress. Psychological resilience is regarded as a personal trait and can be developed in one’s lifetime. Scholars define it as “the role of mental processes and behavior in promoting personal assets and protecting an individual from the potential negative effect of stressors” [8] (p. 16). According to the job demands–resources model, there is an association between job demands and job resources. There are internal psychological factors as well as external corporate factors [10]. Psychological resilience is a typical example of an internal resource.

### 2.3. Employee Resilience

Resilience theory attempts to explain how people overcome negative events through adaptability and flexibility [11]. As mentioned above, psychological resilience is a personal trait. Employee resilience is defined by an employees’ ability to deal with unfavorable situations. This might be affected by an external factor such as organizational resilience and an internal factor such as psychological resilience ([12]).

### 2.4. Organizational Resilience

There are various definitions of organizational resilience. Broadly speaking, the concept of organizational resilience is divided into two main domains: adaption and recovery, and anticipation of an organization [13]. Resilience is “a firm’s ability to effectively absorb, develop situation-specific responses to and ultimately engage in transformative activities to capitalize on disruptive surprises that potentially threaten organization survival” [14] p. 244). Echoed by [15] (p. 61), resilience is “the capability to self-renew over time through innovation.” The second domain is planning and anticipation. Resilience is “the incremental capacity of an organization to anticipate and adjust to the environment” [16] (p. 6) and “prevents budding problems from escalating into a full-blown crisis or breakdown” [15] (p. 431).

Organizational resilience is defined as “the ability to recover from adverse situation by managing existing company resources and capabilities [17] (p. 1219).”

### 2.5. Perceived Mental Well-Being

As defined by [18], “Mental health is a state of well-being in which the individual realizes his or her own abilities, can cope with the normal stresses of life, can work productively and fruitfully, and is able to make a contribution to his or her community” [18] (p. 2), which cannot exist alone, and is interdependent with physical and social functioning.

The enduring political unrest in Hong Kong has brought about significant deterioration in people’s mental health, as supported by findings from a study showing that 41% of participants indicated a subjective decline in mental health due to the social movement [19]. Higher stress levels in young individuals may be attributed to various factors. Firstly, they may have witnessed and/or may have been involved in the conflicts between protesters and the police [19]. Furthermore, another study indicated that during the Anti-Extradition Law Amendment Bill (Anti-ELAB) Movement, tertiary students who were exposed to Anti-ELAB were more prone to Internet addiction, which in turn was associated with depression [20]. A sharp surge in post-traumatic stress disorder (PTSD) symptoms was observed, increasing from 5% to 32% in 2019 [21]. Secondly, family relationships worsened in cases where family members held conflicting political views, leading to compromised social support and mental health. To make matters worse, more than half of the individuals with such problems would seek professional help [21]. Thirdly, because of the socioeconomic downturn associated with COVID-19, people feel increasingly insecure in terms of safety and livelihood. 

## 3. Development of Hypotheses

Psychological resilience could enhance personal well-being [22]. This is because when one can find some resources to cope with adversity, their life will improve as a result [8]. A recent study showed that psychological resilience improved the mental well-being of students during COVID-19 by decreasing academic distress [23]. We propose that perceived mental health is important to workplace employees, particularly in the pandemic era.

**Hypothesis** **1** **(H1).** *Psychological resilience is associated with perceived well-being*.

Resilient individuals would be more flexible in the workplace. It was proposed that emotionally stable employees are able to adapt to new environments and a changing workplace. This relationship is well-supported in the literature [12]. Thus, we proposed that psychological resilience is associated with employee resilience.

**Hypothesis** **2** **(H2).** *Psychological resilience is associated with employee resilience*.

The person-in-situation theory suggests that the behaviors of workers depend on the organizational context [2]. Employees are affected by the organization climate or atmosphere. Individual behaviors are based on the organizational context in which employees with more job security are more committed [24]. 

Organizations could provide employees with more resources to deal with the stress of uncertainty. Organization resilience is an example of an external resource. At the same time, employees become emotionally stable and feel that they are cared for by their employers. 

Thus, we proposed the following:

**Hypothesis** **3** **(H3).** *Organizational resilience is associated with employee resilience*.

**Hypothesis** **4** **(H4).** *Organizational resilience is associated with perceived well-being*.

Perceived well-being comprises “our emotional, psychological, and social well-being”. It affects how we think, feel, and act. It also helps determine how we handle stress, relate to others, and make choices. Mental health is important at every stage of life, from childhood and adolescence through adulthood” [25]. Thus, we proposed the following:

**Hypothesis** **5** **(H5).** *Perceived well-being is associated with employee resilience*.

When employees feel a better sense of well-being, they may be in a better mood and be more devoted to their work. Thus, we proposed the following:

**Hypothesis** **6** **(H6).** *Perceived well-being is associated with work engagement*.

When the organizations facilitate their employees to deal with risks or other uncertainties, employees are more involved in the workplace. As a result, work engagement increases.

**Hypothesis** **7** **(H7).** *Organizational resilience is associated with work engagement*.

Scholars are in consensus that there is an association between employee resilience and work engagement [26]. Employees’ work engagement refers to employees’ commitment to their work organizations. Not only are they expected to fulfill their work role but they are also required to complete their tasks within a short time with minimal costs [27]. Engaged employees are more inclined to form good work relationships and receive adequate social support [28]. A relationship between employee resilience and work engagement in professional information technology staff was found. Researchers concluded that resilient employees have higher confidence at work [3]. In times of uncertainty, higher employee capability may lead to higher work engagement. Thus, we proposed the following:

**Hypothesis** **8** **(H8).** *Employee resilience is associated with work engagement*.

Employees with emotional stability would be more engaged at work. It was found that frontline nurses in China with higher psychological resilience had higher scores in work engagement [29].

**Hypothesis** **9** **(H9).** *Psychological resilience is associated with work engagement*.

**Figure 2:**
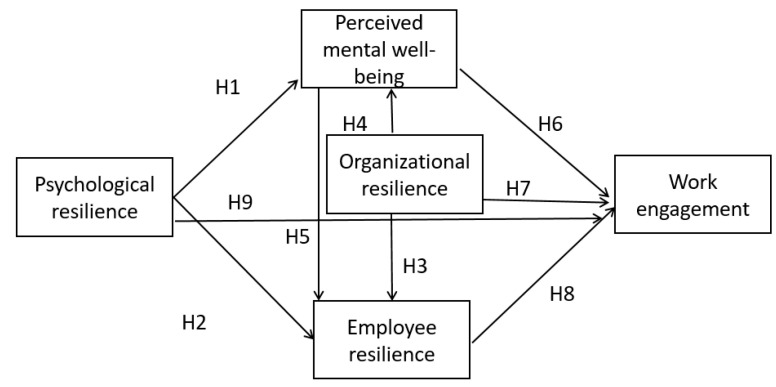
Research model (Source: authors).

Our research model is shown in Figure 2. Perceived mental well-being and employee resilience are hypothesized to be serial mediators in the relationship between organizational resilience and work engagement. Thus we propose the following:

**Hypothesis** **10** **(H10).** *There is a mediation effect between organizational resilience and work engagement. Employee resilience and perceived mental well-being are mediators*.

**Hypothesis** **11** **(H11).** *It is assumed that the mediation effect exists between psychological resilience and work engagement. Employee resilience and perceived mental well-being are mediators*.

## 4. Methodology

### 4.1. Measurement

Measurement items in our research model were all sourced from established scales. Organizational resilience was measured using two dimensions: planned and adaptive resilience using thirteen items [30]. Survey items are listed in Appendix A. Organization resilience was assessed by how an organization plans and adjusts in response to adverse situations. In other words, the organization should act in a proactive manner. Planned resilience refers to preparation before a crisis. Adaptive resilience refers to leadership, linkages, and other abilities. Planned resilience and adaptive resilience measure leadership, creativeness, teamwork, and relationships [31].

There are six items in the Brief Resilience scale measuring psychological resilience [32] and nine items in the Employee Resilience Scale [33] measuring work resilience. Psychological resilience refers to the ability to recover in response to adverse conditions [32]. There are seven items in measuring perceived mental health [34]. The short version of the Warwick–Edinburgh Mental Well-being Scale (SWEMWBS) [35] was adopted for its conciseness and reliability. The SWEMWBS contains 7 statements selected from the 14-item long version of WEMWBS, with 5 frequency options, from ‘none of the time’ to ‘all of the time’. Adequate internal consistency and reliability were demonstrated in a validation study conducted in Norway and Sweden [34]. Work engagement was measured via the Utrecht Work Engagement Scale (UWES-9), which is a 9-item scale with three dimensions of vigor, dedication, and absorption [26].

### 4.2. Data Collection

In 2022, an online survey was conducted with 115 employees from various industries including financial, retail, medical, logistics, hospitality, and education industries. Pilot test was conducted before the main survey to check whether people understood the questionnaire item wordings and meanings. All target respondents were working adults in Hong Kong. Of the total sample, 37.4% were male and 62.6% were female. Of the respondents, 35.7% were aged 18–30 years, 24.3% were aged 31–40 years, and 26.1% were aged 41–50 years. Amongst them, 63.5% were working in large-scale organizations with more than a hundred employees. More than half of the respondents worked in their existing organizations for less than five years and were in entry or supervisory levels. Most of the respondents worked in financial services, medical, education, and professional industries (Table 1). Two-thirds of respondents were working in local companies, and one-third were working in multi-national companies with their headquarters outside Hong Kong, including United States, United Kingdom, other European countries, China, and other Asian countries such as Singapore.

Partial least-squares structural equation modelling was adopted (PLS-SEM because the method does not assume normality in data distribution). The PLS method works well with small sample sizes while covariance-based structural equation modeling requires larger sample sizes. Since our study objective focuses on the prediction of employee resilience and work engagement, PLS-SEM was thus the preferred option [36]. Sample size requirement of minimum path coefficient from 0.21 to 0.3 at 5% significance level was 69. Sample size requirement of minimum path coefficient from 0.21 to 0.3 with 1% significance level was 112. Both sample size requirements were fulfilled by the current study [36]. 

### 4.3. Measurement Model

Measurement model assessments of the six constructs are depicted in Table 2. Nearly all of the indicator loadings fulfilled the recommended minimum threshold of 0.708. One of the indicators for adaptive resilience had a loading of 0.6 and was thus omitted as it did not meet the minimum threshold. Five indicators had loadings close to the thresholds and were hence kept. All constructs had Cronbach’s alpha (0.821 to 0.950) and composite reliability (0.894 to 0.958) values exceeding the recommended thresholds, with satisfactory to good results. All constructs had AVE measures ranging from 0.562 to 0.717, which exceeded the cut-off point of 0.50. In other words, approximately 60% to 70% of the variance of related items was explained by the constructs with satisfactory convergent validity. Lastly, all HTMT values were less than 0.85 (Table 3), suggesting that all constructs were valid and reliable. 

Organizational resilience was a second-order construct. The R-squared values of planned resilience and adaptive resilience were 0.872 and 0.870, respectively, both exceeded the threshold 0.50 of first-order dimensions (Hair et al., 2022). The organizational resilience composite reliability, Cronbach’s alpha, and average variance extracted were 0.931, 0.799, and 0.871, respectively. 

### 4.4. Structural Model

**Figure 3:**
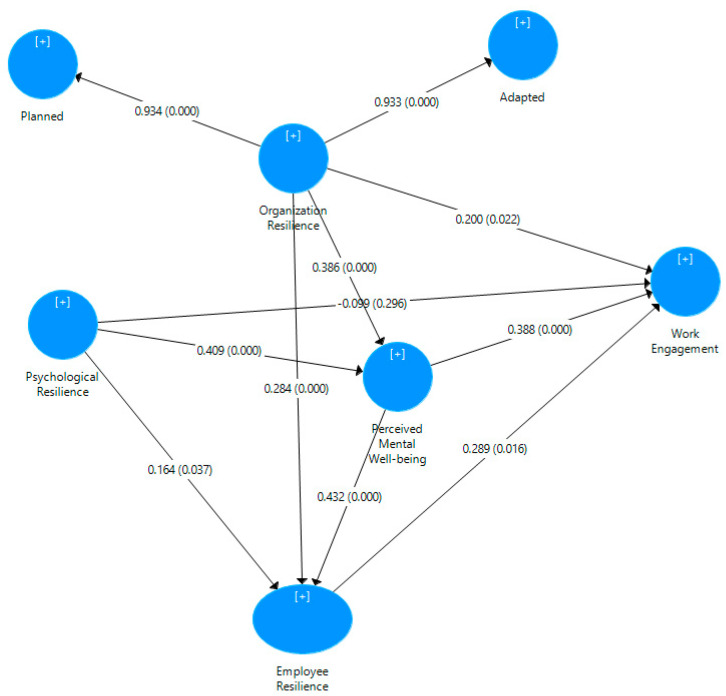
PLS model result.

The structural model demonstrated satisfactory results (Figure 3). The adjusted *R^2^* values of employee resilience, perceived well-being, and work engagement were 0.505, 0.395, and 0.475, respectively, i.e., 39.5% to 50.5% of the variance was explained, suggesting moderate results. Through bootstrap analysis with 5000 subsamples based on the 115 cases, path coefficients and *t*-values were calculated. 

## 5. Results and Discussion

### 5.1. Results of Hypotheses Testing

Work engagement was the outcome of the conceptual model. Organization resilience and psychological resilience were the antecedents. Perceived well-being and employee resilience were the mediators. All the proposed hypotheses were supported except hypothesis nine (Table 4). The direct effect from psychological resilience to work engagement was not supported, i.e., the effects of psychological resilience through two mediators: perceived mental well-being and employee resilience on work engagement were not significant.

Psychological resilience was associated with perceived well-being and employee resilience in multinational companies. Findings suggested that corporate employees adapted easily in response to the rapidly changing local business environment. This was expected since it has been more than two years since the COVID-19 outbreak started. Our results concur with those of a study conducted in Christchurch, 2016, for the two association (Hypotheses 1 and 2) [12]. Their target respondents were tourism organization owners, which represent the employer perspective. In contrast, our study respondents are employees, which provide another angle on the proposed relationship. 

The strength of the relationship between psychological resilience and employee resilience is weaker compared to that found by Ref [12]. We proposed that employee resilience is also subject to the influence of organizational resilience.

Organizational resilience was associated with perceived well-being and employee resilience for all companies, which aligned with our hypothesis as foreign and local companies are all aware of the importance of organization resilience. Companies provided relevant resources, measures, and support such as flexible working hours or work from home arrangement during COVID-19. Unlike a sudden single event such as an earthquake, a pandemic situation becomes an issue for the employers or organization. Thus, corporate efforts have been put in place and our study provides empirical support for the argument. 

Employee resilience and perceived well-being were associated with work engagement. Similar results were obtained in a recent study [28]. Their findings indicate that the strength of a relationship is greater than the direct effect from organizational resilience. Companies should allocate more resources to enhance employee resilience and perceived well-being.

### 5.2. Mediation Effect

**Figure 4:**
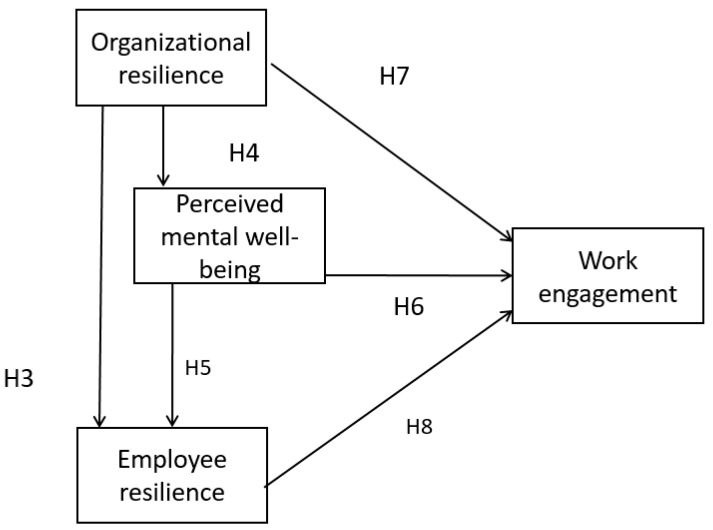
Mediation Analysis model.

There was a partial mediation effect linking organization resilience and work engagement with perceived well-being as a mediator (Hypotheses 4 and 6). The direct effect of organization resilience on work engagement (Hypothesis 7) was significant, meaning that perceived well-being was a partial mediator on the outcome variable of work engagement. Similarly, there was a partial mediation effect linking organization resilience and work engagement with employee resilience as a mediator (Hypotheses 3 and 8), indicating that employee resilience was also a partial mediator in this mediation analysis. Finally, perceived well-being and employee resilience were serial mediators. The association between organization resilience and perceived well-being (Hypothesis 4), association between perceived well-being and employee resilience (Hypothesis 5) and association between employee resilience and work engagement (Hypothesis 8) were significant. This is a typical example involving serial and parallel mediation, which the authors consider a complex mediation analysis involving two or more parallel mediation effects at the same time, also known as multiple mediation analysis [36] (Figure 4). Hypothesis 10 was supported with partial mediation. 

**Figure 5:**
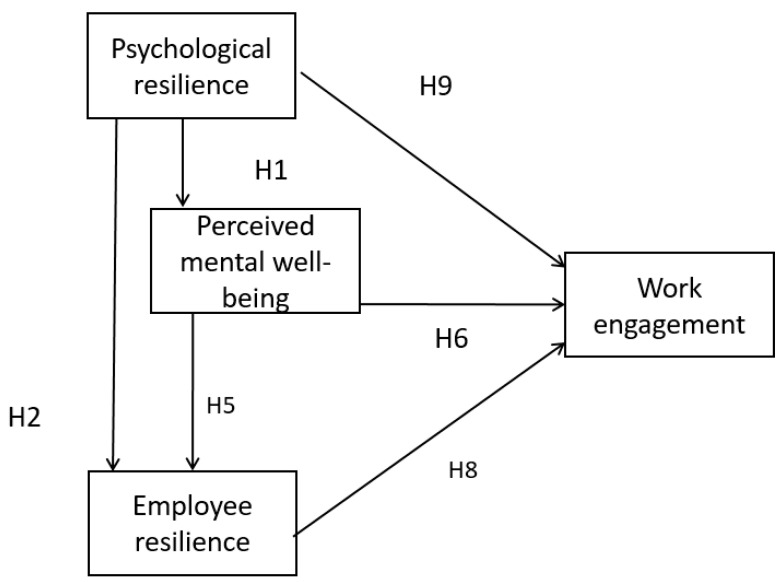
Complex mediation model.

There was a full mediation effect linking psychological resilience and work engagement. Parallel mediation effects were observed in our research model. First, the direct effect of psychological resilience and work engagement was not significant (Hypothesis 9). Second, when perceived well-being was a mediator, the association between psychological resilience and perceived well-being (Hypothesis 1) and association between perceived well-being and work engagement (Hypothesis 6) were significant. Third, when employee resilience was a mediator, the association between psychological resilience and employee resilience (Hypothesis 2) and association between employee resilience and work engagement (Hypothesis 8) were significant. Finally, it was found that both perceived well-being and employee resilience were serial mediators. Moreover, the association between psychological resilience and perceived well-being (Hypothesis 1), association between perceived well-being and employee resilience (Hypothesis 5), and association between employee resilience and work engagement (Hypothesis 8) were significant. This presents another example of complex mediation analysis involving serial and parallel mediation (Figure 5). Hypothesis 11 was supported with full mediation.

### 5.3. Theoretical Contributions

This study bears several theoretical contributions. First, a basic conceptual framework of employee resilience was proposed using internal and external factors based on the research of Herrman et al. [6]. Our study provides important perspectives from the internal stakeholder and the employee. They know the organization situation very well and are probably affected most by organization policy. Second, we provided empirical evidence that supported the research model showing how organizational resilience and psychological resilience affect employee resilience through the mediator of perceived mental well-being. Finally, two complex mediation models were presented using a combination of serial and parallel mediations. Starting variables are organizational resilience and psychological resilience, respectively. Work engagement is the final outcome variable.

### 5.4. Managerial Implications

There are some practical implications. More measures need to be devised to enhance psychological resilience, and organizational resilience, since these variables affect employee resilience followed by work engagement.

Recommended measures to strengthen organization resilience and enhance the mental health and resilience of employees include resource commitment, information flow, resource integration, local responsiveness, and flexibility of control. Resources are labor, material and financial capital. Information flow among subsidiaries and headquarters are important. Information blocking increases the expenses of information. Companies work with business partners such as suppliers, regulators and competitors on the integration of resources. Local responsiveness and flexibility of control applies to the subsidiaries [37].

Similarly, measures could be implemented to enhance psychological resilience. These measures maintain good mood or emotions in employees and regulate negative emotions. Sports activities and Yoga courses could be organized. Professional support and help from counsellors are to be sought on an on-demand basis [38].

## 6. Conclusions

The purpose of this study was to examine the effects of organizational resilience and psychological resilience on work engagement in the workplace. It was found that the direct effect of organizational resilience on work engagement was significant and psychological resilience on work engagement was insignificant. Organizational resilience and psychological resilience were associated with perceived well-being and employee resilience. Employee resilience and perceived well-being were associated with work engagement. Employee resilience and perceived well-being were found to be mediators. Complex mediation models were identified. This study explained the underlying mechanism of how organizational resilience and psychological resilience affect employees’ work engagement.

However, this study is not without limitations. First, this is a cross-sectional study and hence causality claims cannot be made. It would be more ideal to employ a longitudinal research design. Second, there may also be some other factors that affect work engagement such as teamwork and a sense of belonging [38]. Control variables may be introduced in further research works. Finally, employers’ perspectives could be studied instead of employees’ perspectives.

## Figures and Tables

**Table 1:** Demographic data of respondents.

Category		Frequency	Percentage %
Gender	Male	43	37.4
	Female	72	62.6
Age	18–30	41	35.7
	31–40	28	24.3
	41–50	30	26.1
	51–60	13	11.3
	61 or above	3	3.0
Size	Less than 5 persons	8	7.0
	5–20 persons	16	13.9
	21–50 persons	9	7.8
	51–100 persons	9	7.8
	101 or above persons	73	63.5
Tenure	Less than 6 months	10	8.7
	6 months to less than 2 years	31	27
	2 years to less than 5 years	32	27.8
	5 years to less than 10 years	17	14.8
	10 years or above	25	21.7
Level	Entry	52	45.2
	Supervisory	26	22.6
	Middle management	23	20
	Senior management	9	7.8
	Director	5	4.3
Industry	Tourism	2	1.7
	Financial services	21	18.3
	Trading and logistics	13	11.3
	Construction	1	0.9
	Information Technology	7	6.1
	Engineering	5	4.3
	Surveyor and Property management	1	0.9
	Professional services, education, medical services	47	40.9
	Cultural and creative	4	3.5
	Others	14	12.2

**Table 2:** Measurement Model Assessment.

Construct	Item	Loading	Cronbach’s Alpha	Composite Reliability	AVE
Psychological Resilience	PR1 PR2 PR3 PR4	0.655 0.774 0.877 0.915	0.821	0.884	0.659
Employee Resilience	ER1 ER2 ER3 ER4 ER5 ER6 ER7	0.777 0.841 0.851 0.805 0.816 0.663 0.826	0.905	0.925	0.638
Planned Resilience	PR1 PR2 PR3 PR4	0.700 0.873 0.872 0.867	0.849	0.899	0.691
Adaptive Resilience	AR1 AR2 AR4 AR5	0.767 0.833 0.854 0.841	0.842	0.894	0.680
Perceived Well-being	WB1 WB2 WB3 WB4 WB5 WB6 WB7	0.734 0.864 0.631 0.834 0.691 0.692 0.774	0.868	0.899	0.562
Work Engagement	WE1 WE2 WE3 WE4 WE5 WE6 WE7 WE8 WE9	0.859 0.878 0.934 0.823 0.763 0.874 0.903 0.776 0.792	0.950	0.958	0.717

**Table 3:** Assessing Discriminant Validity (HTMT).

Constructs	1	2	3	4	5	6
1. Adapted Resilience						
2. Employee Resilience	0.582					
3. Planned Resilience	0.869	0.606				
4. Psychological Resilience	0.291	0.528	0.328			
5. Perceived well-being	0.540	0.727	0.570	0.599		
6. Work Engagement	0.586	0.634	0.566	0.309	0.676	

**Table 4:** Results of hypotheses testing.

Hypothesis	Path	(*β*) Path Coefficient	*t*-Value	*p*-Value	Result
H1	Psychological Resilience >> Perceived well-being	0.409	5.003	<0.001 ***	Supported
H2	Psychological Resilience >> Employee Resilience	0.164	2.088	0.037 *	Supported
H3	Organization Resilience >> Employee Resilience	0.284	4.181	<0.001 ***	Supported
H4	Organization Resilience >> Perceived well-being	0.386	5.326	<0.000 ***	Supported
H5	Perceived well-being >> Employee Resilience	0.432	5.138	<0.000 ***	Supported
H6	Perceived well-being >> Work Engagement	0.388	3.892	<0.000 ***	Supported
H7	Organization Resilience >> Work Engagement	0.200	2.232	0.026 *	Supported
H8	Employee Resilience >> Work Engagement	0.289	2.400	0.016 *	Supported
H9	Psychological Resilience >> Work engagement	−0.099	1.053	0.292	Unsupported

(Bootstrap samples = 5000, *n* = 115 cases) * *p* < 0.05; *** *p* < 0.001.

## Data Availability

Data are available upon request.

## Appendix A

**Table A1:** Modified survey items.

Construct Name and Abbreviation	Items
Psychological Resilience (PR)	I tend to bounce back quickly after hard times. I have a hard time making it through stressful events. It is hard for me to snap back when something bad happens. I tend to take a long time to get over set-backs in my life.
Employee Resilience (ER)	I successfully manage a high workload for long periods of time. I resolve crises competently at work. I learn from mistakes at work and improve the way I do my job. I re-evaluate my performance and continually improve the way I do my job. I effectively respond to feedback at work, even criticism. I seek assistance to work when I need specific resources. I use change at work as an opportunity for growth.
Planned Resilience (P)	Given how others depend on us, the way we plan for the unexpected is appropriate. Our organization is committed to practicing and testing its emergency plans to ensure they are effective. We have a focus on being able to respond to the unexpected. We have clearly defined priorities for what is important during and after a crisis.
Adaptive Resilience (AR)	People in our organization are committed to working on a problem until it is resolved. Our organization maintains sufficient resources to absorb some unexpected change. If key people were unavailable, there are always others who could fill their role. There would be good leadership from within our organization if we were struck by a crisis. We are known for our ability to use knowledge in novel ways.
Mental well-being (WB)	I have been feeling optimistic about the future I have been feeling useful I have been feeling relaxed I have been dealing with problems well I have been thinking clearly I have been feeling close to other people I have been able to make up my own mind about things
Work Engagement (WE)	At my work, I feel bursting with energy
	At my job, I feel strong and vigorous I am enthusiastic about my job My job inspires me
	When I get up in the morning, I feel like going to work
	I feel happy when I am working intensely
	I am proud of the work that I do
	I am immersed in my work
	I get carried away when I am working

## References

[1] Wut T.M., Xu J., Wong S.-M. (2021). Crisis management research (1985–2020) in the hospitality and tourism industry: A review and research agenda. Tour. Manag..

[2] Hirst G., Van Knippenberg D., Chen C.-H., Sacramento C.A. (2011). How Does Bureaucracy Impact Individual Creativity? A Cross-Level Investigation of Team Contextual Influences on Goal Orientation–Creativity Relationships. Acad. Manag. J..

[3] Malik P., Garg P. (2017). Learning organization and work engagement: The mediating role of employee resilience. Int. J. Hum. Resour. Manag..

[4] Soanes C., Stevenson A. (2006). Oxford Dictionary of English.

[5] Luthar S., Cichetti D., Becker B. (2000). The Construct of Resilience: A Critical Evaluation and Guidelines for Future Work. Child Dev..

[6] Herrman H., Stewart D., Diaz-Granados N., Berger E., Jackson B., Yuen T. (2011). What is Resilience?. Can. J. Psychiatry.

[7] Tugade M.M., Fredrickson B.L. (2004). Resilient Individuals Use Positive Emotions to Bounce Back From Negative Emotional Experiences. J. Personal. Soc. Psychol..

[8] Fletcher D., Sarkar M. (2013). Psychological Resilience. Eur. Psychol..

[9] Tonkin K., Malinen S., Naswall K., Kuntz J.C. (2018). Building employee resilience through wellbeing in organizations. Hum. Resour. Dev. Q..

[10] Demerouti E., Bakker A.B., Nachreiner F., Schaufeli W.B. (2001). The job demands resources model of burnout. J. Appl. Psychol..

[11] Senbeto D., Hon A. (2020). Market turbulence and service innovation in hospitality: Examining the underlying mechanisms of employee and organizational resilience. Serv. Ind. J..

[12] Prayag G., Spector S., Orchiston C., Chowdhurry M. (2020). Psychological resilience, organizational resilience and life satisfaction in tourism firms: Insights from the Canterbury earthquakes. Curr. Issues Tour..

[13] Duchek S. (2020). Organizational resilience: A capability-based conceptualization. Bus. Res..

[14] Lengnick-Hall A., Beck T., Lengnick-Hall M. (2011). Developing a capacity for organizational resilience through strategic human resource management. Hum. Resour. Manag. Rev..

[15] Boin A., Eeten M. (2013). The Resilient Organization-A critical appraisal. Public Manag. Rev..

[16] Ortiz-de-Mandojana N., Bansal P. (2016). The long-term benefits of organizational resilience through sustainable business practices. Strateg. Manag. J..

[17] Chowdhury M., Prayag G., Orchiston C., Spector S. (2019). Postdisaster social capital, adaptive resilience and business performance of tourism organizations in Christchurch, New Zealand. J. Travel Res..

[18] World Health Organization (WHO) (2019). Universal Health Coverage for Mental Health. https://apps.who.int/iris/bitstream/handle/10665/310981/WHO-MSD-19.1-eng.pdf?sequence=1&isAllowed=y.

[19] Ng R. (2020). Mental Health in Hong Kong: Its Current Status and Collective Responses from Mental Health Professionals. Psychiatr. Times.

[20] Tang G., Hung E.P.W., Au-Yeung H.-K.C., Yuen S. (2020). Politically Motivated Internet Addiction: Relationships among Online Information Exposure, Internet Addiction, FOMO, Psychological Well-being, and Radicalism in Massive Political Turbulence. Int. J. Environ. Res. Public Health.

[21] Ting V. (2020). Hong Kong Protests: Mental Health Issues Rise Drastically with more than 2 Million Adults Showing Signs of Post-Traumatic Stress Disorder, Study Finds. South China Morning Post.

[22] Pooly J.A., Cohen L. (2010). Resilience: A definition in context. Aust. Community Psychol..

[23] Rasheed N., Fatima I., Tariq O. (2022). University students’ mental well-being during COVID-19 pandemic: The mediating role of resilience between meaning in life and mental well-being. Acta Psychol..

[24] Mischel W., Mangusson D., Endler N.S. (1977). The interaction of person and situation. Personality at the Crossroads: Current Issues in International Psychology.

[25] United States Government (2020). What Is Mental Health. https://www.mentalhealth.gov/basics/what-is-mental-health.

[26] Cooke F., Cooper B., Bartram T., Wamg J., Mei H. (2019). Mapping the relationships between high-performance work systems, employee resilience and engagement: A study of the banking industry in China. Int. J. Hum. Resour. Manag..

[27] Schaufeli W.B., Bakker A.B., Salanova M. (2006). The measurement of Work Engagement with a short questionnaire. Educ. Psychol. Meas..

[28] Fredrickson L., Tugade M., Waugh E., Larkin R. (2003). What good are positive emotions in crisis? A prospective study of resilience and emotions following the terrorist attacks on the United States on 11 September 2001. J. Personal. Soc. Psychol..

[29] Lyn H., Yao M., Zhang D., Liu X. (2020). The relationship among Organizational Identity, Psychological Resilience and Work Engagement of the First-Line Nurses in the Prevention and Control of COVID-19 based on structural equation model. Risk Manag. Healthc. Policy.

[30] Orchiston C., Prayag G., Brown C. (2016). Organizational resilience in the tourism sector. Ann. Tour. Res..

[31] Lee A.V., Vargo J., Seville E. (2013). Developing a tool to measure and compare organizations’ resilience. Nat. Hazards Rev..

[32] Smith B.W., Dalen J., Wiggins K., Tooley E., Christopher P., Bernard J. (2008). The brief resilience scale: Assessing the ability to bounce back. Int. J. Behav. Med..

[33] Kuntz J.R., Malinen S., Naswall K. (2017). Employee resilience: Directions for resilience development. Consult. Psychol. J. Pract. Res..

[34] Stewart-Brown S., Tennant A., Tennant R., Platt S., Parkinson J., Weich S. (2009). Internal construct validity of the Warwick-Edinburgh Mental Well-being Scale (WEMWBS): A Rasch analysis using data from the Scottish Health Education Population Survey. Health Qual. Life Outcomes.

[35] Tennant R., Hiller L., Fishwick R., Platt S., Joseph S., Weich S., Parkinson J., Secker J., Stewart-Brown S. (2007). The Warwick-Edinburgh Mental Well-being Scale (WEMWBS): Development and UK validation. Health Qual. Life Outcomes.

[36] Hair J.F., Hult G.T.M., Ringle C., Sarstedt M. (2022). A Primer on Partial Least Squares Structural Equation Modeling (PLS-SEM).

[37] Cao M., Alon I. (2021). Overcoming the liability of foreignness–A new perspective on Chinese MNCs. J. Bus. Res..

[38] Wut T.M., Lee S., Xu J. (2022). Work from Home Challenges of the Pandemic Era in Hong Kong: A Stimulus-Organism-Response Perspective. Int. J. Environ. Res. Public Health.

